# Targeted opening of the blood-brain barrier using VCAM-1 functionalised microbubbles and “whole brain” ultrasound

**DOI:** 10.7150/thno.93172

**Published:** 2024-07-02

**Authors:** Vanessa A. Johanssen, Jia-Ling Ruan, Oliver Vince, Alec Thomas, Sarah Peeters, Manuel Sarmiento Soto, Jessica Buck, Michael Gray, Eleanor Stride, Nicola R. Sibson

**Affiliations:** 1 Department of Oncology, University of Oxford, Oxford, UK.; 2 Institute of Biomedical Engineering, Department of Engineering Science, University of Oxford, UK.; 3 Nuffield Department of Orthopaedics, Rheumatology and Musculoskeletal Research, University of Oxford, UK.

**Keywords:** therapeutic ultrasound, blood-brain barrier, microbubbles, targeted drug delivery, metastases

## Abstract

Metastatic tumours in the brain now represent one of the leading causes of death from cancer. Current treatments are largely ineffective owing to the combination of late diagnosis and poor delivery of therapies across the blood-brain barrier (BBB). Conjugating magnetic resonance imaging (MRI) contrast agents with a monoclonal antibody for VCAM-1 (anti-VCAM1) has been shown to enable detection of micrometastases, two to three orders of magnitude smaller in volume than those currently detectable clinically. The aim of this study was to exploit this targeting approach to enable localised and temporary BBB opening at the site of early-stage metastases using functionalised microbubbles and ultrasound.

**Methods:** Microbubbles functionalised with anti-VCAM1 were synthesised and shown to bind to VCAM-1-expressing cells *in vitro*. Experiments were then conducted *in vivo* in a unilateral breast cancer brain metastasis mouse model using Gadolinium-DTPA (Gd-DTPA) enhanced MRI to detect BBB opening. Following injection of Gd-DTPA and targeted microbubbles, the whole brain volume was simultaneously exposed to ultrasound (0.5 MHz, 10% duty cycle, 0.7 MPa peak negative pressure, 2 min treatment time). T1-weighted MRI was then performed to identify BBB opening, followed by histological confirmation via immunoglobulin G (IgG) immunohistochemistry.

**Results:** In mice treated with targeted microbubbles and ultrasound, statistically significantly greater extravasation of Gd-DTPA and IgG was observed in the left tumour-bearing hemisphere compared to the right hemisphere 5 min after treatment. No acute adverse effects were observed. There was no investigation of longer term bioeffects owing to the nature of the study.

**Conclusion:** The results demonstrate the feasibility of using targeted microbubbles in combination with low intensity ultrasound to localise opening of the BBB to metastatic sites in the brain. This approach has potential application in the treatment of metastatic tumours whose location cannot be established *a priori* with conventional imaging methods.

## Introduction

Metastases are the leading cause of cancer-related deaths worldwide 1 and metastatic spread to the brain is a significant clinical problem. Brain metastases are the most prevalent of intracranial malignancies 2 and the median survival for untreated patients is only 5 weeks 3,4. Incidence varies with primary tumour type, with the most common primaries to metastasise to the brain being breast, lung, and melanoma. Currently, surgery is the preferred treatment for accessible, symptomatic lesions in patients with controlled extracranial disease and a good performance status 5, and the addition of radiotherapy can increase local control 5-7. However, local tumour recurrence is a persistent problem, occurring in up to 40% of cases 5, and in their 2014 World Cancer Report, the World Health Organisation identified the lack of targeted therapies specific for brain metastases as one of the major unmet clinical needs in global cancer care 8

Critically, in the early stages of development, when treatment would be most effective, brain metastases grow behind an intact blood-brain barrier (BBB), which very effectively prevents both their detection with imaging contrast agents and treatment with systemic agents. Only very small or gaseous molecules can pass through the BBB with ease 9-11. For molecules to passively diffuse through the BBB, they generally must have high lipid solubility, low polar surface area and low molecular weight 12. Moreover, the components of the BBB continuously adapt in response to various physiological changes in the brain 13, resulting in further challenges to drug delivery, such as upregulation of multiple trans-membrane efflux pumps 14-17.

A number of strategies has been developed to overcome the problem of an intact BBB^18^-^24^, but these all suffer one or more limitations, including transient windows of permeability (< 1 h), size-restricted access (< 1 kDa), lack of targeting to tumour sites, dose-limiting toxicity or the need to know tumour location. We have, however, recently reported one promising approach that utilizes the local inflammatory response of the brain vascular endothelium in the presence of micrometastases and, specifically, local upregulation of the tumour necrosis factor (TNF) type 1 receptor. By targeting this receptor with a mutant TNF protein, we have shown that it is possible to achieve an extended window (≥ 24 h) of permeabilization specifically at sites of brain micrometastases, which allows entry of agents across a therapeutically relevant size range (0.5-150 kDa) and which targets all metastatic tumours within the brain with no requirement for *a priori* knowledge of location 25,26. Whilst the above approach will facilitate entry of circulating therapeutics, an alternative approach would be to achieve targeted local delivery of a therapeutic agent contained within a permeabilizing entity itself. One approach that has the potential to achieve this aim is the combination of microbubbles and ultrasound.

Widely used as an imaging modality for over 50 years, ultrasound has also now been shown to have extensive therapeutic applications, including delivery of genes, chemotherapeutic agents and oncolytic viruses, increasing membrane permeability to molecules and enhancing transport of drugs across the BBB 27. A large proportion of these bio-effects are induced or enhanced by acoustic cavitation. 28. Early work demonstrated temporary disruption of the BBB with the combination of ultrasound and microbubbles (US-BBBD) 29, and showed repeatable BBB opening without damage to surrounding tissue 30. As this technique is not specific to the therapeutic agent delivered, it has the potential to improve the treatment of a wide range of brain diseases and disorders by allowing free passage of molecules up to 2000 kDa into the brain 31. Several clinical trials using US-BBBD have already taken place with promising results 32,33, and further trials are either active or recruiting for a range of indications including glioblastoma (GBM), Alzheimer's disease, amyotrophic lateral sclerosis (ALS) and Parkinson's disease.

Whilst US-BBBD represents a promising therapeutic technique for a wide range of cerebral pathologies, the need to know the exact target location in the brain prior to treatment represents a major barrier, particularly to the treatment of metastatic tumours in the brain that are undetectable until sufficiently large to disrupt the BBB. Actively targeting micrometastases by using microbubbles designed to bind specifically and selectively to tumour associated vessels is one possible solution to this challenge. As discussed above, we have previously demonstrated that the vascular endothelium closely associated with brain micrometastases is activated and expresses a number of molecules involved in the inflammatory cascade. One such molecule is vascular cell adhesion molecule 1 (VCAM-1), which is upregulated on the luminal surface of the vascular endothelium very early in brain metastasis formation 34. We have previously demonstrated that targeting VCAM-1 with an iron-oxide based MRI contrast agent enables the presence of brain micrometastases to be detected in the presence of an intact BBB 34,35. The aim of this study, therefore, was to adapt this approach and assess the feasibility of developing ultrasound-responsive microbubbles targeted to VCAM-1 to facilitate localised US-BBBD. If successful, this approach has the potential to enable targeted focal delivery of therapeutics contained within the microbubbles themselves in the early stages of micrometastasis development, without the need for *a priori* knowledge of location, and removing the need for complex focusing or craniotomy to compensate for distortion of the ultrasound field by the skull.

## Materials and Methods

### Microbubble preparation

1,2-dibehenoyl-sn-glycero-3-phosphocholine (DBPC), 1,2-distearoyl-sn-glycero-3-phosphoethanolamine-N-[biotinyl(polyethylene glycol)-2000], 1,2-distearoyl-sn-glycero-3-phosphoethanolamine-N-[amino(polyethylene glycol)-2000] and 1,2-distearoyl-sn-glycero-3-phosphoethanolamine-N-(7-nitro-2-1,3-benzoxadiazol-4-yl) were purchased from Avanti Polar Lipids Inc. (Alabaster, AL, USA). Streptavidin, avidin, and propylene glycol were purchased from Sigma-Aldrich (Gillingham, Dorset, UK). Phosphate buffered saline (PBS) and Alexa Fluor 647 anti-His Tag Antibody were from ThermoFisher Scientific (Milton Park, OXON, UK). Sulphur hexafluoride (SF6) was purchased from the BOC Group PLC (Woking, SURREY, UK) and perfluorobutane (PFB) from F2 Chemicals Ltd. (Preston, LANCS, UK). The polyhistidine-tagged Mouse VCAM-1 protein (50163-M08H) is purchased from Sino Biological (Beijing, China).

The ratios of the different components used are shown in Table 1 and a schematic overview of the different microbubble formulations used can be found in the Supplementary Material (Figure S1).

The anti-VCAM-1 conjugated microbubbles (AV-MBs) were prepared using a biotin-streptavidin-biotin bridging methodology. AV-MB manufacture began with the preparation of biotinylated microbubbles (biot-MBs). DBPC, DSPE-PEG2000 and DSPE-PEG2000-Biotin were mixed in a glass vial in the quantities given in Table 1. For AV-MBs used *in vitro*, DSPE-NBD was also added at this point. The mixture was heated on a hotplate at 58 ºC for 12 h, to allow for the chloroform in which the lipids are supplied to evaporate to form a lipid film. This lipid film was suspended in 5 mL of PBS for 1 h on a hotplate at 85 ºC under constant magnetic stirring. Lipids were then dispersed for 90 s using a sonicator (XL 2000, probe diameter 3 mm, 20 W, 22.5 kHz, Misonix, Farmingdale, NY, US) with the tip completely immersed in the lipid-PBS solution (power setting 4). The glass vial was then placed in an 85 °C water bath for 60 s to keep the solution above the melting point of the lipids. The solution was then sonicated for a further 30 s at power setting 4 to ensure homogeneous dispersion. Biot-MBs were then formed by placing the sonicator tip at the air-water interface under constant gas (SF6 or PFB) flow and sonicating for 30 s (power setting 14). Immediately after production, the vial containing the biot-MB suspension was capped and placed in ice for at least 20 min.

To ensure efficient and uniform binding of the streptavidin to the biot-MBs, it was important to first remove the free DSPE-PEG2000-Biotin from solution. This was done via centrifugal washing as follows. (1) Biot-MBs were added to a 10 mL syringe (BD life sciences, US). This syringe was then capped and placed inside a 50 mL centrifuge tube. (2) The biot-MBs were then centrifuged at 350 g for 5 min at 4°C. (3) The syringe plunger was then gradually lowered to remove the subnatant, leaving only the biot-MB cake in the syringe. (4) The biot-MBs were then re-suspended by drawing 2 mL PBS into the syringe. Steps 2 to 4 were repeated for each washing step for three times. To incubate microbubbles with the next solution (e.g. streptavidin or antibody), the 2 mL PBS in Step 4 was replaced with the desired solution.

To generate microbubbles coated with streptavidin, biot-MBs were washed three times to remove excess biotinylated lipid in solution. After the third centrifugal wash, the cake of biot-MBs was re-suspended in a solution at 4°C made from 2 mL PBS and 0.2 mg streptavidin (ThermoFisher Scientific). This mixture was mixed gently for 15 min using a roller shaker (Cole-Palmer, St. Neots, CAM, UK).

To generate microbubbles coated with anti-VCAM-1, strept-MBs were washed three times to remove excess streptavidin in solution (not bound to the microbubble surface). After the third centrifugal wash, the cake of strept-MBs was re-suspended in a solution at 4°C made from 1.2 mL PBS and 0.8 mL Rat Anti-Mouse CD106-BIOT at 0.5 mg/mL in PBS/NaN_3_, (Southern Biotech, Birmingham, AL, USA). This mixture was mixed gently for 30 min using a roller shaker (Cole-Palmer). These microbubbles were then washed a final time to remove excess antibody. IgG coated microbubbles (IgG-MBs) were prepared using the same method as AV-MBs, except the antibody used was Rat IgG1-BIOT at 0.5 mg/mL in PBS (0116-08, Southern Biotech). Please note that for the reported *in vivo* experiments, avidin was substituted for streptavidin owing to its lower immunogenicity, but the manufacturing protocols were unchanged.

### Microbubble size and concentration measurements

For quantification of microbubble size and concentration for the *in vitro* experiments, 10 μL of the microbubble suspension was transferred onto a Neubauer improved cell counting chamber (Hausser Scientific Company, Horsham, PA, USA) under a 24 mm × 24 mm glass coverslip (VWR International, Lutterworth, LEICS, UK). At least 10 images of microbubbles were acquired at 40 x magnification using a Leica DM500 microscope (Leica Microsystems GmbH, Germany) coupled with a CCD camera (MicroPublisher 3.3 RTV, QImaging, Surrey, BC, Canada). Microbubble sizing and counting was performed using a purpose-written code in MATLAB® (The MathWorks Inc., Natick, MA, US) 36. For the *in vivo* experiments, microbubbles were passed through an 18 G blunt filter needle (with 5 μm filter, 305211) three times to remove large bubbles, then sized and counted using a Contador Coulter Multisizer 4e (Beckman Coulter, High Wycombe, BUCKS, UK).

### Verification of streptavidin conjugation to MBs

Streptavidin Pacific-Blue (ThermoFisher Scientific) was used to verify that the streptavidin conjugation strategy resulted in uniform and consistent coating of streptavidin on the strept-MBs. Strept-MBs were manufactured as above, with the streptavidin being replaced with 0.2 mg streptavidin Pacific-Blue to form strept-PB-MBs. These strept-PB-MBs were then centrifugally washed once to remove excess Pacific-Blue from solution and 10 μl were then placed between a glass microscope slide (ThermoFisher Scientific) and a glass coverslip (VWR International). They were imaged in the fluorescence channels of NBD (green, excitation/emission maxima ~463/536 nm), Pacific Blue (blue, excitation/emission at 410/455 nm) and brightfield on a Nikon Eclipse Ti microscope (Nikon UK, Surbiton, SURREY, UK). NBD and Pacific-Blue fluorescence images were then overlaid to enable visual verification of streptavidin-Pacific Blue binding on each MB. As a control in this experiment, the binding of streptavidin Pacific-Blue to non-biotinylated microbubbles was also tested using the same method.

### Verification of anti-VCAM-1 conjugation to MBs

To verify the uniform conjugation of anti-VCAM-1 to AV-MBs, AV-MBs were incubated with a solution of fluorescent VCAM-1 protein at 4°C for 60 min to form AV-647-MBs. This solution was prepared by incubating 10 ng of polyhistidine-tagged Mouse VCAM-1 protein with 10 ng of Alexa Fluor 647 anti-His Tag Antibody in 2 mL PBS at 4°C for 30 min. These AV-647-MBs were then centrifugally washed once to remove excess Alexa Fluor-647 from solution and, as for the strept-PB-MB above, 10 µl were placed between two glass coverslips (VWR International, US) and imaged in the fluorescence channels of NBD (green, excitation/emission maxima ~463/536 nm) and Alexa Fluor 647 (red, excitation/emission at 650/665 nm) on a Zeiss LSM 780 confocal microscope (Carl Zeiss AG, Aalen, Germany). NBD and Alexa Fluor 647 fluorescence images were then overlaid to enable visual verification of anti-VCAM binding on each MB. Again, the binding of Alexa Fluor 647 to IgG-MBs was also tested using the same method as a control.

### *In vitro* targeting assay

MBs were manufactured on the day of each experiment and stored at 4°C until immediately prior to use. MB concentrations were matched to remove the impact of any inconsistencies arising from the MB manufacturing process. b.End3 mouse brain endothelial cells (Sigma Aldrich) were maintained in DMEM Ham-F12 medium with 10% FBS and 2 mM L-glutamine. Cells (6 × 10^6^ cells/mL) were seeded in 35 mm μ-Dishes or μ-Slides (Ibidi GmbH, Grafelfing, Germany) for the static flotation and flow testing, respectively. After 24 hours, the culture medium was removed and replaced with fresh medium, either with or without lipopolysaccharide (LPS, 10 μg/mL); exposure to LPS increases the expression of VCAM-1 37. Binding experiments were conducted at least 20 hours post LPS stimulation.

For static flotation testing, antibody conjugated MB binding to LPS-stimulated endothelial cells was tested using static flotation. A 2 mL solution of either AV-MBs or IgG-MBs (at 5 × 10^7^ per mL) in PBS was added to a 35 mm μ-Dish (Ibidi, Germany) containing cells. The dish was then inverted and placed on a shaker (Cole Palmer, UK) for 5 min. This allowed the MBs to float into contact with the cells and ensured gentle mixing. After the incubation, the cells were washed twice gently with PBS, then incubated for 5 min with 1 μl of CellMask Deep Red plasma membrane stain in 1 mL PBS. After a further 2 gentle washes with PBS, dual fluorescence microscopy (Nikon Eclipse Ti) was used to visualise any MBs (using the NBD stain in the MB shell) still on the cell membrane (using the CellMask stain). ImageJ software was then used to threshold each image and count the number of pixels that were cells and MBs, rather than background. MB-cell binding efficiency for each condition was then calculated by dividing the number of green (MB) pixels by the number of red (cell) pixels.

For flow testing, the ability of antibody conjugated MBs to bind to LPS-stimulated cells and stay bound under flow was tested using μ-Slides containing b.End3 endothelial cells. A 3 mL solution of either AV-MBs or IgG-MBs (at 1.25 x 10^8^ per mL) was loaded into a syringe (BD Life Science, US). This syringe was then connected via Teflon tubing to a μ-Slide channel and placed in a syringe pump. The flow rate of the syringe pump was calculated such that the wall shear stress experienced by the cells was 10 Dynes/cm (1 Pa, the highest shear stress expected to occur *in vivo* 38). The cells were then gently washed twice with PBS using the protocol recommended by the μ-Slide manufacturer to remove any MBs still in suspension, before the addition of CellMask Deep Red plasma membrane stain at a 1000 times dilution in PBS. After allowing 5 min for the CellMask incubation, the cells were washed gently twice more. As with the static flotation testing, dual fluorescence microscopy (Eclipse Ti, Nikon, US) was then used to visualise any MBs (using the NBD stain in the MB shell) still on the cell membrane (using the CellMask stain). These images were then processed in the same way using ImageJ 432 software.

### *In vivo* mouse brain metastasis experiments

Animal experiments were carried out in accordance with the University of Oxford Policy Use of Animals in Scientific Research and the ARRIVE Guidelines under Project Licence number PC4932237 and were approved by the University of Oxford Clinical Medicine Ethics Review Committee and the UK Home Office (Animals [Scientific Procedures] Act 1986).

To determine the time-courses of VCAM-1 expression and natural BBB breakdown, female BALB/c mice (6-10 weeks old, Charles River, UK) were injected intracerebrally into the left striatum with 5 x 10^3^ 4T1-GFP mouse mammary carcinoma cells. 4T1 tumours naturally metastasise to the brain when a primary tumour is implanted into the mammary fat pad, and brain metastases can also be induced haematogenously by intracardiac or intracarotid injection of 4T1 cells, which is considered to be a close representation of the natural route of seeding to the brain. In all of the above cases, however, metastases will seed randomly throughout the brain. In the context of the current experimental design, it was important to know where the tumours were seeded and to retain the contralateral hemisphere as a non-tumour bearing control. We have previously demonstrated that intracerebral injection of metastatic breast cancer cells leads to tumour growth that is very similar to that found when metastases are induced via intracardiac injection, as well as to human brain metastasis growth 39. Consequently, the intracerebrally induced model can be considered to be representative of metastasis growth once seeded within the brain, and, as such, was used here in this proof-of-principle experiment study. It should be noted that the study was not designed to fully mimic brain metastasis in all its various forms, but rather to test the ability of VCAM-1-targeting to focus ultrasound mediated BBBD at tumour sites specifically. For VCAM-1 expression, mice were perfusion-fixed at day 5, 10 or 21 (n = 5 per timepoint*). To assess BBB breakdown, animals were imaged at day 7 (n = 10), 14 (n = 6), 21 (n = 6), 28 (n = 17) or 35 (n = 9).

To assess the effects of targeted microbubbles and ultrasound on BBB permeability, female BALB/c mice (7-8 weeks old, Charles River, UK) (n = 9, 17-21 g) were injected intracerebrally with 4T1-GFP mouse mammary carcinoma cells, as described above. Fourteen days after the tumour cells were injected, when the tumour is known to have VCAM-1 upregulation, yet is still small enough for the BBB to remain mostly intact, the mice underwent the targeted microbubble and ultrasound protocol. On the day of microbubble and ultrasound treatment, mice were maintained under anaesthesia with isoflurane (1.5-2.0%) in 50% O2:50% air. Hair was removed from the top of the skull and the chest with clipping followed by depilatory hair removal cream, for unobstructed ultrasound application.

### Magnetic resonance imaging

For all MRI studies, mice were anaesthetised with 1-3% isoflurane in 30% O_2_:70% N_2_ or air and positioned in a 26 mm inner diameter transmit/receive birdcage RF coil (Rapid Biomedical GmbH, Germany) using a homebuilt cradle. Mouse temperature was monitored and maintained at 37 ± 0.5 °C with a rectal probe and heating blanket feedback system. Respiration was monitored using a pressure balloon.

To determine the time-course of natural BBB breakdown, animals were imaged in a 9.4-T Agilent DDR MRI spectrometer (Agilent Technologies Inc., USA) using a T1-weighted spin-echo multi-slice (SEMS) sequence with the following parameters: repetition time (TR) = 500 ms; echo time (TE) = 20 ms; number of averages = 2; field of view (FOV) = 20 × 20 mm; acquisition matrix = 256 × 256; in-plane resolution = 78 μm isotropic; slice thickness 1 mm; number of slices = 8. Mice were imaged both pre- and 5 min post-intravenous injection of 30 μL of Gd-DTPA (Omniscan, GE Healthcare, Amersham, BUCKS, UK) to assess BBB breakdown. Areas of gadolinium enhancement were manually segmented using ITK-SNAP version 3.6.0.

**Figure 1:**
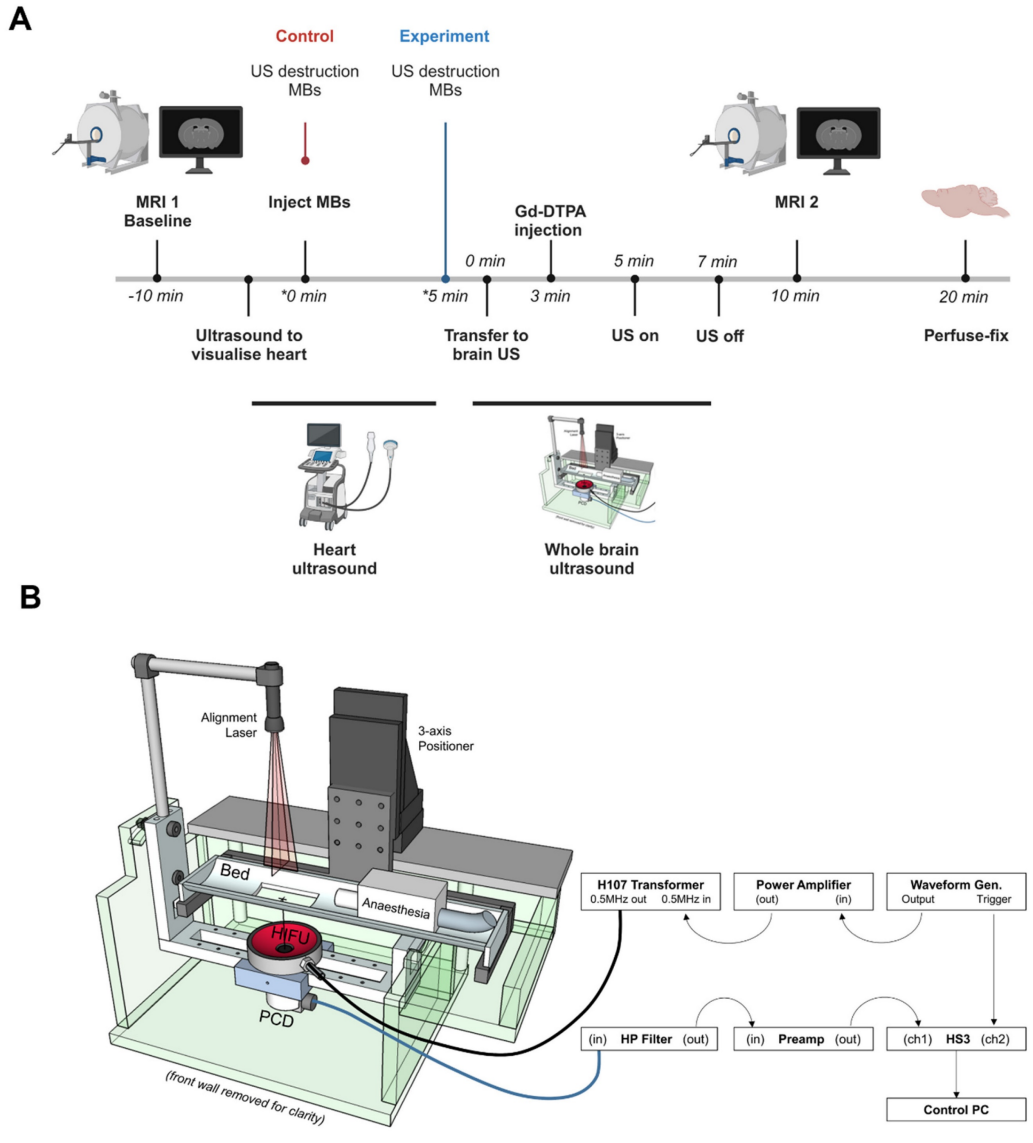
** Timeline (A) and Schematic of the experimental set up (B) used for *in vivo* ultrasound exposure of a mouse model of breast cancer brain metastasis.** VCAM-1 targeted microbubbles were destroyed by ultrasound to the heart, immediately upon injection for the control group, whereas in the experimental group, ultrasound destruction at the heart was performed 5 min later to destroy any circulating VCAM-1 microbubbles not bound to the target (panel A was created with Biorender®).

To assess the effect of targeted microbubbles on BBB breakdown, animals were imaged in a 7.0 T horizontal bore magnet (Agilent Technologies Inc., US). As above, all mice were imaged pre- and post-intravenous injection of 30 μL of Gd-DTPA (Omniscan, GE Healthcare, Amersham, BUCKS, UK)/microbubble and ultrasound exposure using a T1-weighted spin-echo multi-slice (SEMS) sequence, with the following parameters: repetition time (TR) = 500 ms; echo time (TE) = 8 ms; number of averages = 4; FOV = 22.5 × 22.5 × 22.5 mm; acquisition matrix = 128 × 128 × 20; in-plane resolution = 175 μm isotropic; slice thickness 0.7 mm; number of slices = 20. Comparison of the pre- and post-treatment MRI scans allowed quantitation of Gd-DTPA uptake in the brain parenchyma. A summary of this timeline can be found in Figure 1A.

### Ultrasound imaging and microbubble destruction protocol

Following the first imaging session, prior to microbubble and ultrasound treatment, mice were moved to a Vevo 3100 preclinical ultrasound imaging setup (with MH250 transducer, FUJIFILM VisualSonics). Coupling gel was applied to the chest and ultrasound applied to image the heart in nonlinear contrast mode (frequency = 18 MHz, transmit power = 25%, acquisition frame rate = 5 Hz, dynamic range = 40 dB). Upon obtaining a clear image of the left ventricle, 200 µl microbubbles (~10^8^/mL) were slowly injected intravenously, via a pre-implanted cannula. To destroy any unbound circulating microbubbles, burst mode was used. Based on the microbubble characterisation data and preliminary experiments with commercial contrast agents and non-functionalised microbubbles, it was decided that the same microbubble formulation (i.e. AV-MB) needed to be used for all *in vivo* experiments. This was to avoid there being any differences in bubble size distribution, concentration, acoustic response and/or circulation kinetics that might affect BBBD and, thus, render the experiment unfair. Instead, the primary comparison of BBBD was made between the left and right sides of the brain before and after ultrasound exposure in individual mice. Thus, all of the animals would be exposed to the same microbubbles and the same ultrasound field, and differences in BBB opening determined by whether or not bubbles were preferentially bound in the activated region. A further comparison was made between animals in which AV-MB were allowed to circulate for 5 min before destruction in the heart, to allow for binding in the brain (“Experiment” group) and animals in which the burst mode was applied immediately post-injection so that AV-MB would make fewer passes of the circulation and hence be expected to bind to a much lower extent unless they were simply mechanically lodged (“Control” group).

### Ultrasound treatment protocol

Ultrasound exposure to the brain was carried out using a purpose-built setup (Figure 1B) that allowed reproducible treatment of mice, whilst in an MRI cradle and in a stereotactic frame, and thus, enabling consistent positioning for comparative analysis, as described previously 40. Briefly, animals were held in position in the cradle using cheek bars for the duration of ultrasound treatment so that the whole brain volume could be consistently exposed. A 500 kHz single element transducer (H107, Sonic Concepts, US), mounted directly below a holder for the MRI cradle so that the focal volume (ellipsoid with lateral and axial full-width half-amplitude dimensions in water of 4.1 and 25.2 mm, respectively as measured using a needle hydrophone, Precision Acoustics, Dorset, UK) was aligned prior to the experiments by maximizing the reflection signal from an alignment target located at the position where the mouse's head would be positioned. Animals were inverted to allow ultrasound to pass through the top of the skull and coupled to the mylar window using degassed ultrasound gel. As discussed previously, attenuation and distortion of the field due the transmission path were expected to be within the measurement uncertainty of the hydrophone.

### Acoustic emissions monitoring

Acoustic emissions produced during the *in vivo* experiments were captured using a single element unfocused transducer (Panametrics, V382-SU, Olympus Industrial Systems Europa, Southend-on-Sea, Essex, UK) as a passive cavitation detector (PCD) with a 3.5 MHz centre frequency co-aligned with the ultrasound transducer. A HS3 digital oscilloscope (TiePie, The Netherlands) and the PCD were triggered with a function generator. The PCD signal was first passed through a 500 kHz notch filter to remove the fundamental. Each recorded voltage trace from the PCD was then truncated in the time domain to remove the portion of the signal prior to the first anatomic reflection. A Hanning window was applied to the voltage trace to reduce edge effects. Matlab® (Mathworks, US) was used to compute the fast Fourier transform (FFT) and the power spectral density (PSD) for each voltage trace. The harmonic and ultraharmonic signal powers were determined by integrating the power spectral density with respect to frequency over 30 kHz bands at each harmonic and ultraharmonic from 1 to 8 MHz. The broadband power was determined by integrating the power spectral density with respect to frequency over the remaining frequencies between 1 and 8 MHz. The total energy of acoustic emissions for each sample was calculated by integrating the powers (harmonic, ultraharmonic and broadband) with respect to time.

### MRI analysis of gadolinium extravasation into the parenchyma

To analyse differences in BBB breakdown, the mean intensity on the post- versus pre-treatment scans was measured and compared between hemispheres. MR images were analysed in ImageJ v.1.52p software. The striatum of the left and right hemispheres was manually selected. The selected area was measured and the mean intensity per mm^3^ was calculated.

### Histological analysis of VCAM-1 expression and serum IgG extravasation

At the assigned time-points for VCAM-1 expression or immediately after the microbubble experiment, mice were perfusion-fixed with periodate lysine paraformaldehyde (PLP) containing 0.025% (w/v) glutaraldehyde (PLP_light_) and the brains extracted. Mouse brains were fixed overnight in PLP_light_, then immersed in a 30% sucrose solution until they sank. Brains were embedded in Tissue-Tek® O.C.T, frozen in isopentane and stored at -20 °C until required. Each brain was cryosectioned into 20 μm slices, collecting the region of the brain that contains the striatum (tumour implantation area) using a Leica cryostat (model CM1950). Slides were air dried overnight then stored at -20 °C until analysis.

VCAM-1 immunohistochemistry was performed using a rat anti-VCAM-1 antibody (Southern Biotech, AL, USA) 1:100, followed by biotinylated goat and rat secondary 1:100. Biotinylated anti-mouse IgG (Vector Labs, UK) was utilised to identify the extravasation of serum IgG into the brain parenchyma. Analysis of VCAM-1 and IgG staining intensity was carried out with Aperio Image Scope (Leica Biosystems, Germany) software, as described previously. The region of the left hemisphere where the tumour cells could be visualised was manually marked. The contralateral hemisphere was manually marked to be the mirror image of the tumour areas. Pixels with a value of 0-175 in the red channel (corresponding to the areas with dark DAB staining) were considered to be locations of serum IgG extravasation. These pixels are referred to as IgG positive. The total number of IgG positive pixels per mm^2^ of brain was calculated for each cerebral hemisphere.

### Statistical analysis

Statistical analysis was performed using built-in analysis methods in Prism v. 10.0.0 software (GraphPad Software, San Diego, CA, USA). Data with error bars are shown as mean +/- standard deviation (SD). One-tailed Wilcoxon matched-pairs test was used for statistical comparisons. P values were used to assess significance of the data where *p<0.05, **p<0.01.

## Results & Discussion

### Microbubble characteristics

**Figure 2:**
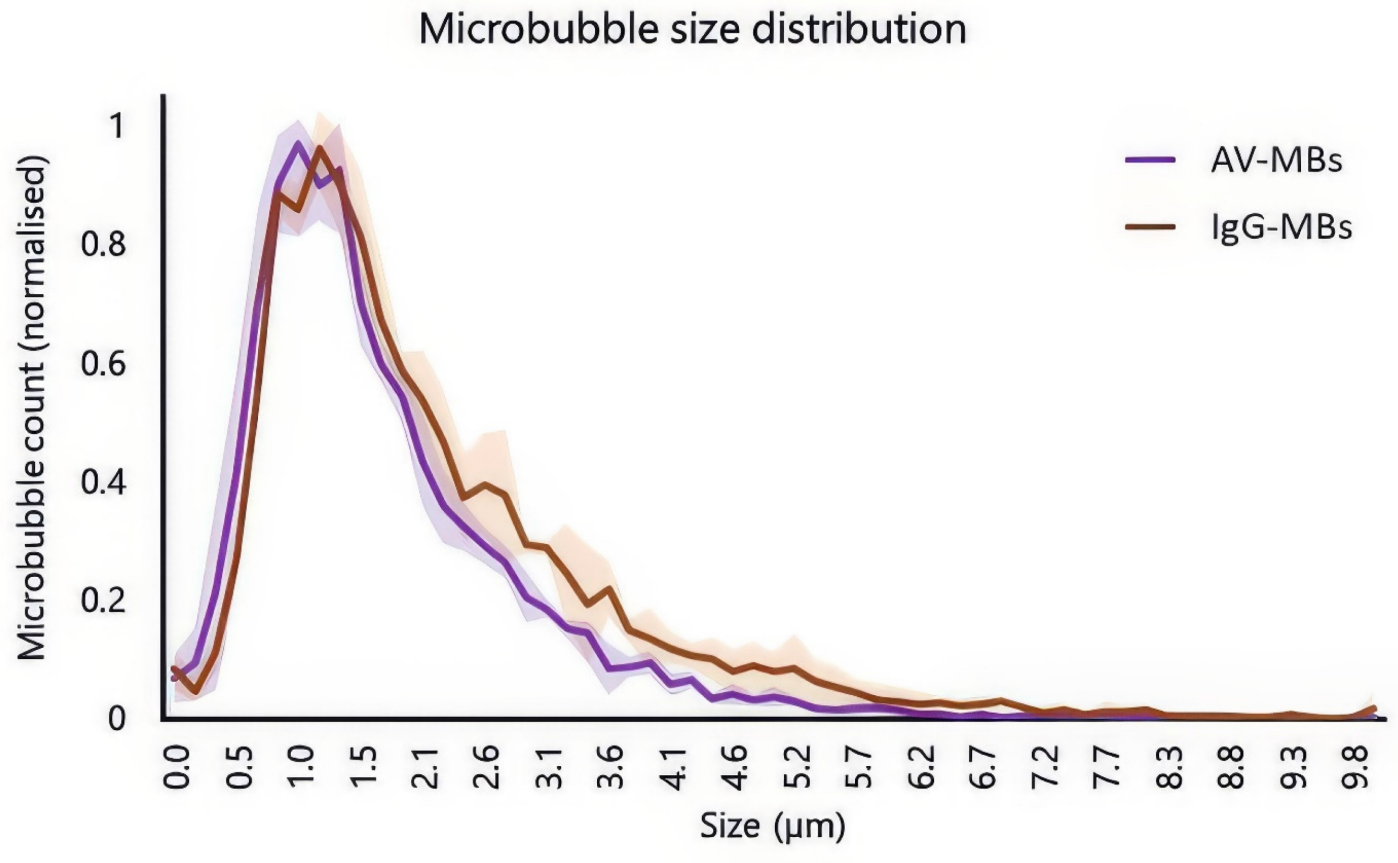
**Size distributions and schematic of the different microbubble formulations used in the experiments**. Solid line shows the mean average of 3 independent samples of AV-MBs and IgG-MBs. The shaded area indicates 1 standard deviation.

Figure 2 shows the normalised average size distributions of AV-MBs and IgG-MBs used experimentally. There was a slightly higher proportion of IgG-MBs in the 2.6 to 5.7 μm range when compared to the AV-MBs and this may have had a small influence on MB targeting as larger MBs experience greater viscous drag forces in fluid flow. However, there was no significant difference between the mean and modal sizes of these MB distributions and so the size distributions were not expected to materially impact the targeting results.

### Verification of streptavidin and anti-VCAM-1 conjugation

A uniform coating of Pacific-Blue was observed on all strept-PB-MBs, in contrast to non-biotinylated MBs that had been incubated with streptavidin Pacific-Blue and washed equally (Figure S2A). This indicated successful conjugation. Similarly, to validate the conjugation of anti-VCAM-1 antibodies, AV-MBs and IgG-MBs were incubated separately with a solution of Alexa Fluor 647 labelled VCAM-1 protein. A uniform coating of Alexa Fluor 647 was observed on all AV-MBs, in contrast to IgG-MBs for which no Alexa Fluor 647 fluorescence was observed (Figure S2B).

### Static flotation testing

Under static flotation, some IgG-MBs were seen to bind non-specifically to the membranes of LPS-stimulated cells but the binding of AV-MBs was much higher (Figure S3). Some AV-MBs also bound to non-stimulated cells, but this was to be expected due to the fact that non-stimulated cells are not fully quiescent. There was found to be a slight difference between the confluence of the cell samples at the time of testing, but no correlation between the MB-cell binding and the cell confluence was observed (Figure S4A-B).

### Flow testing

**Figure 3:**
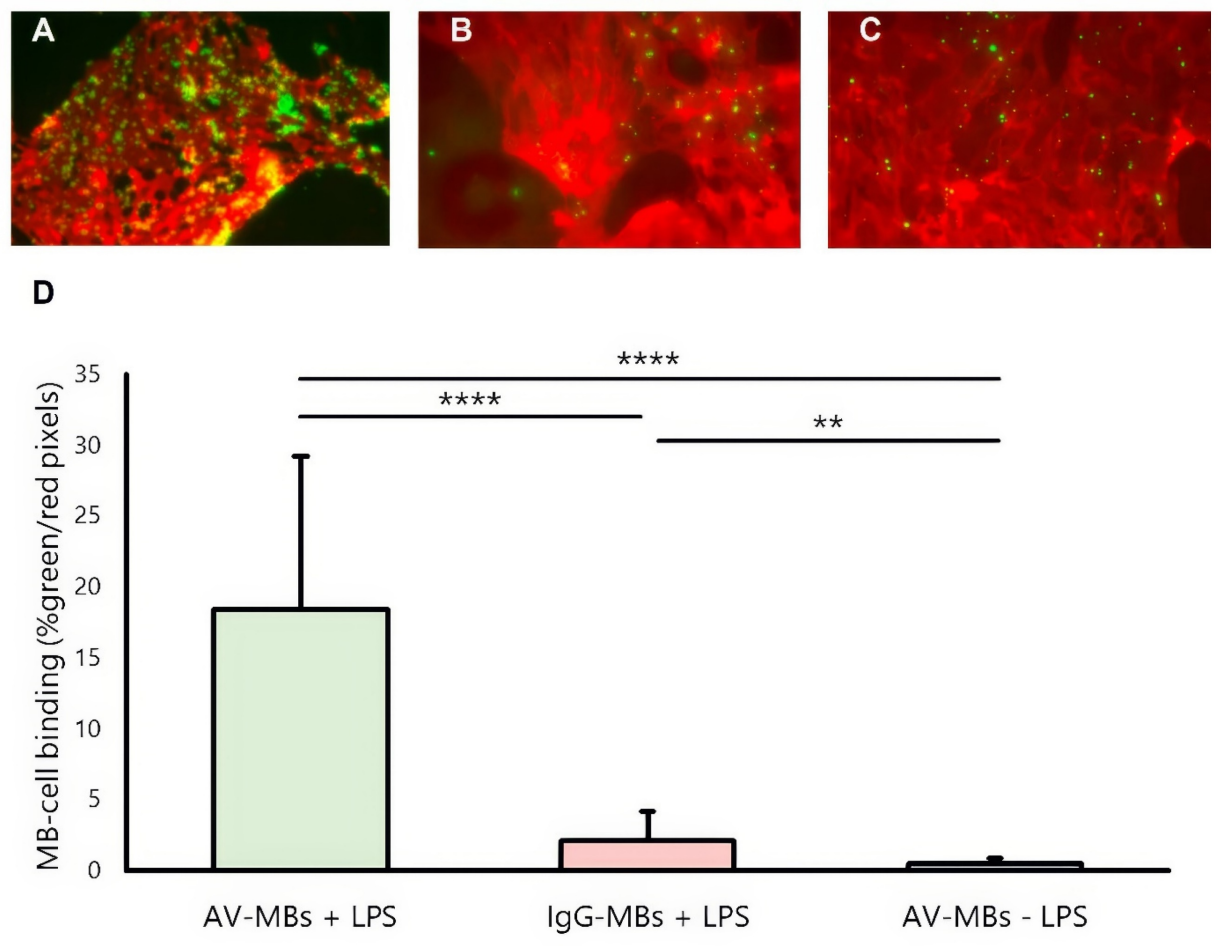
** MB-cell binding under flow.** (A-C) Representative dual fluorescence microscopy images showing the AV-MBs and IgG-MBs bound to B.end3 endothelial cells exposure under flow (D). The binding of AV-MBs and IgG-MBs to B.end3 endothelial cells. Binding percentage was calculated by the proportion of each dual fluorescence microscopy frame occupied by microbubbles divided by the proportion of the frame occupied by cells (n = 5 per group).

Figure 3 shows representative dual-fluorescence microscopy images of MBs (green, from the NBD in the MB shell) and cells (from the CellMask Deep Red plasma membrane stain). Again, visibly more AV-MBs (Figure 3A) bound to stimulated cells than IgG-MBs (Figure 3B), and this is again more than the number of AV-MBs that bind to unstimulated cells (Figure 3C). These results are corroborated by the quantification shown in Figure 3D. In contrast to the results in static flotation testing, there was minimal binding of AV-MBs to unstimulated cells when under flow. Again, there was no obvious relationship between the cell confluence and the MB-cell binding (Figure S4C-D). The small amount of non-specific binding observed is consistent with previous findings, as even microbubbles that have no surface functionalisation have been found to show small degrees of binding to cells *in vitro*. We have previously shown that one explanation may be lipid transfer between the microbubble coating and cell membrane 41 and there may be other mechanisms, e.g. most cells have some phagocytic capacity.

### Determination of timepoint for testing in mouse brain metastasis model

In previous studies, we have identified VCAM-1 as an early-stage marker of micrometastatic sites in the brain and the application of VCAM-1 targeting for both MRI detection of brain micrometastases and targeted therapy (34,42-46. The 4T1-GFP mouse model of breast carcinoma has been routinely used to investigate breast cancer metastasis to the brain. We first assessed expression of vascular expression of VCAM-1 over time to determine a suitable timepoint for the microbubble experiments. VCAM-1 upregulation on cerebral vessels associated with the 4T1-GFP tumour was identified both immunohistochemically and by immunofluorescence (Figure S5A). Quantitatively, both the overall area of VCAM-1 expression (Figure S5B) and the percentage of vessels expressing VCAM-1 (Figure S5C) increased over time, with approximately 50% of vessels expressing VCAM-1 by day 10 post-implantation and peaking at approximately 70% of blood vessels 21 days post-implantation. Next, we assessed BBB permeability over time in order to determine the point at which natural BBB breakdown occurs. T1w MRI showed marked gadolinium contrast enhancement by day 35 post-implantation, with some enhancement at day 28 (Figure S6A-B). From the above results, day 14 was chosen as the timepoint for experiments herein, as there is VCAM-1 upregulation for the targeted microbubbles, but minimal natural BBB breakdown.

**Figure 4:**
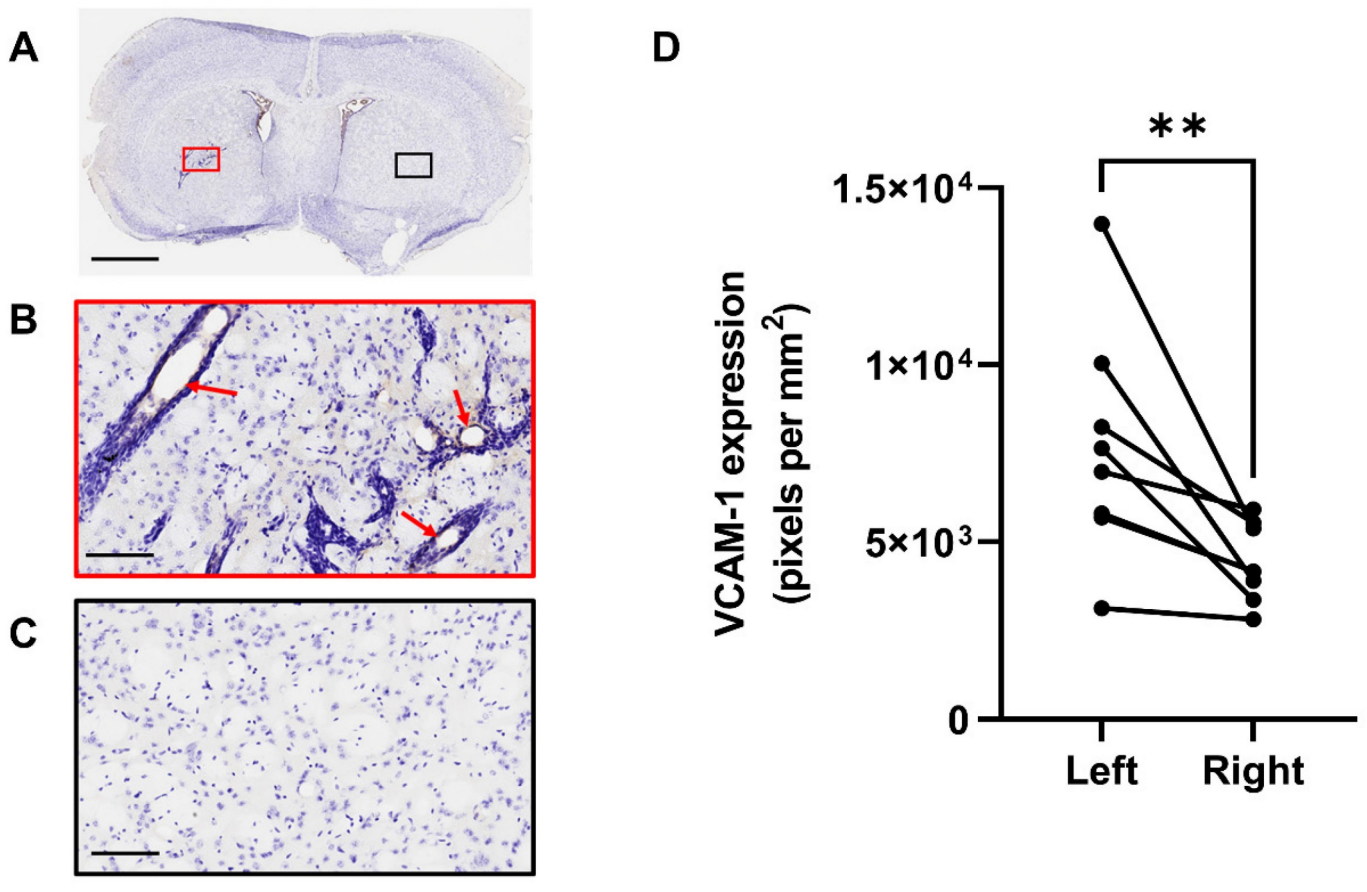
**Quantification of VCAM-1 expression at day 14.** A) VCAM-1 (brown) immunohistochemistry results of mouse brain sections at day 14 post tumour cell implantation. B) Activated VCAM-1 was detected on the vessels of the left (tumour) hemisphere (red arrows). C) Minimal VCAM-1 expression was found on the vessels of the right (control) side. D) Quantification of VCAM-1 expression, left versus right hemispheres, in animals that were in study at day 14. Sections counterstained with cresyl violet; scale bars = 2 mm (A) and 100 µm (B,C).

Figure 4 shows VCAM-1 immunohistochemistry in the tumour hemisphere (Figure 4A red box and 4B) and in the control, contralateral hemisphere (Figure 4A black box and 4C) at the day 14 time-point. Analysis of VCAM-1 expression by positive pixel account demonstrated a significant increase in the left hemisphere compared to the right (Figure 4D). Importantly, constitutive VCAM-1 expression was negligible outwith the tumour site, consistent with what is observed in human brain metastases 44.

### MRI detection of microbubble/US-induced BBB breakdown in mouse brain metastasis model

**Figure 5:**
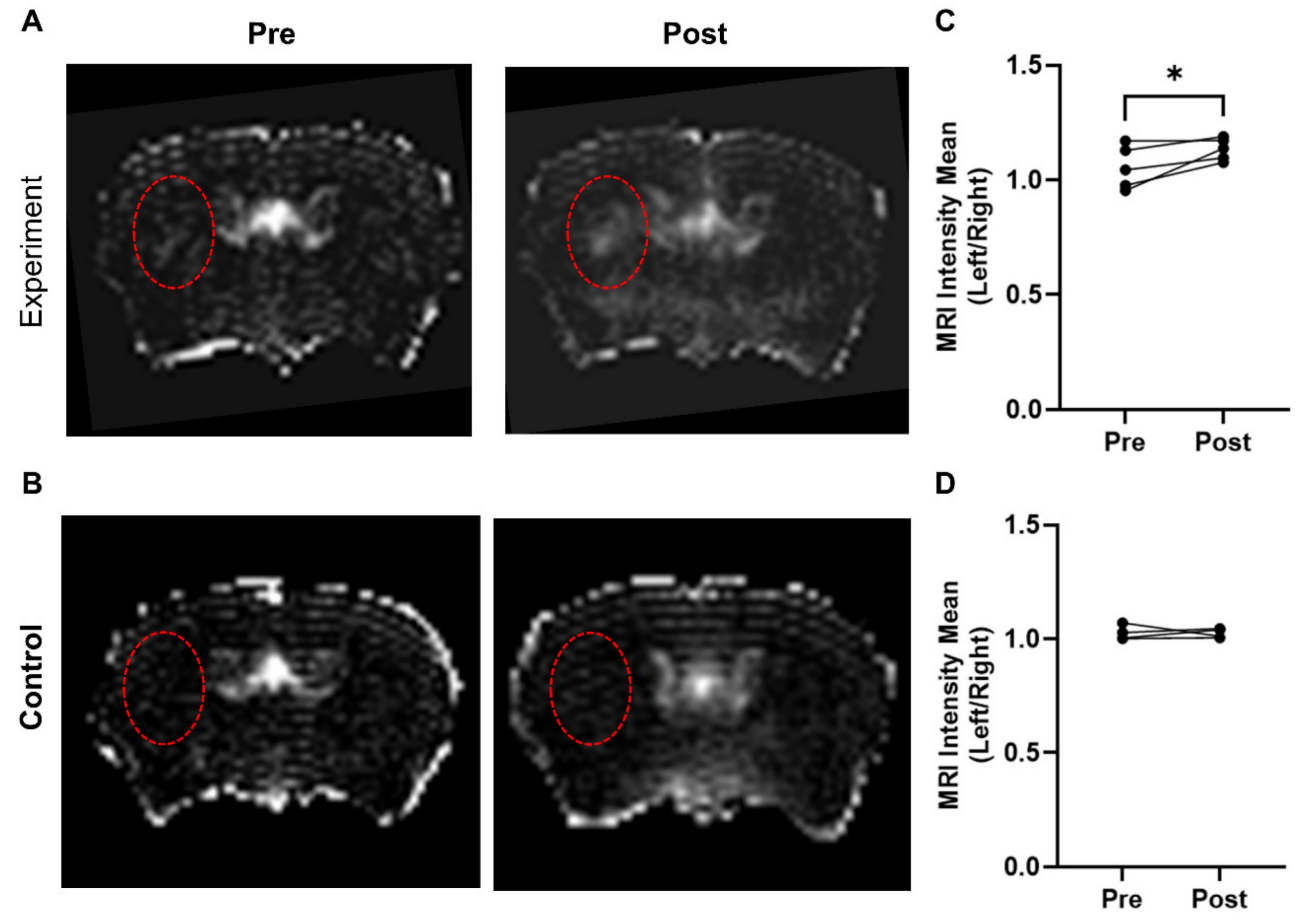
** MRI detection of microbubble/US-induced BBB breakdown.** Representative T1-weighted, Gd-DTPA contrast images of mouse brain pre and post AV-MB injection and ultrasound treatment with microbubble destruction either 5 mins later (experiment group) (A) or destroyed immediately (control group) (B). Red dashed circles highlight tumour location in the striatum. The experiment group showed significant Gd-DTPA uptake post AV-MB and ultrasound treatment in the tumour area when compared to the contralateral area (C) *p<0.05. No significant change was observed in the control group (D).

In the experimental group (AV-MBs destructed after 5 mins), a visible difference was evident between the left and right hemispheres (Figure 5A), particularly close to the tumour site. No significant difference was observed in the control group (VCAM-1-MBs destroyed immediately after injection; Figure 5B). In both groups, although to a greater extent in the experimental group, some smaller, non-specific areas of breakdown were evident, particularly in the sinuses and draining veins. Quantitation of the left-to-right ratio of mean signal intensity in the striatum pre- and post-treatment revealed that only the experimental group showed a significant difference (Figure 5C-D). The energy of acoustic emissions was measured in all experiments. Unfortunately, there was a high degree of variability in the results (Figure S7), likely due to anatomical differences and extensive scattering of the signal. Consequently, it was not possible to distinguish any consistent features to compare with the MRI results.

### Immunohistochemical detection of microbubble/US-induced BBB breakdown

**Figure 6:**
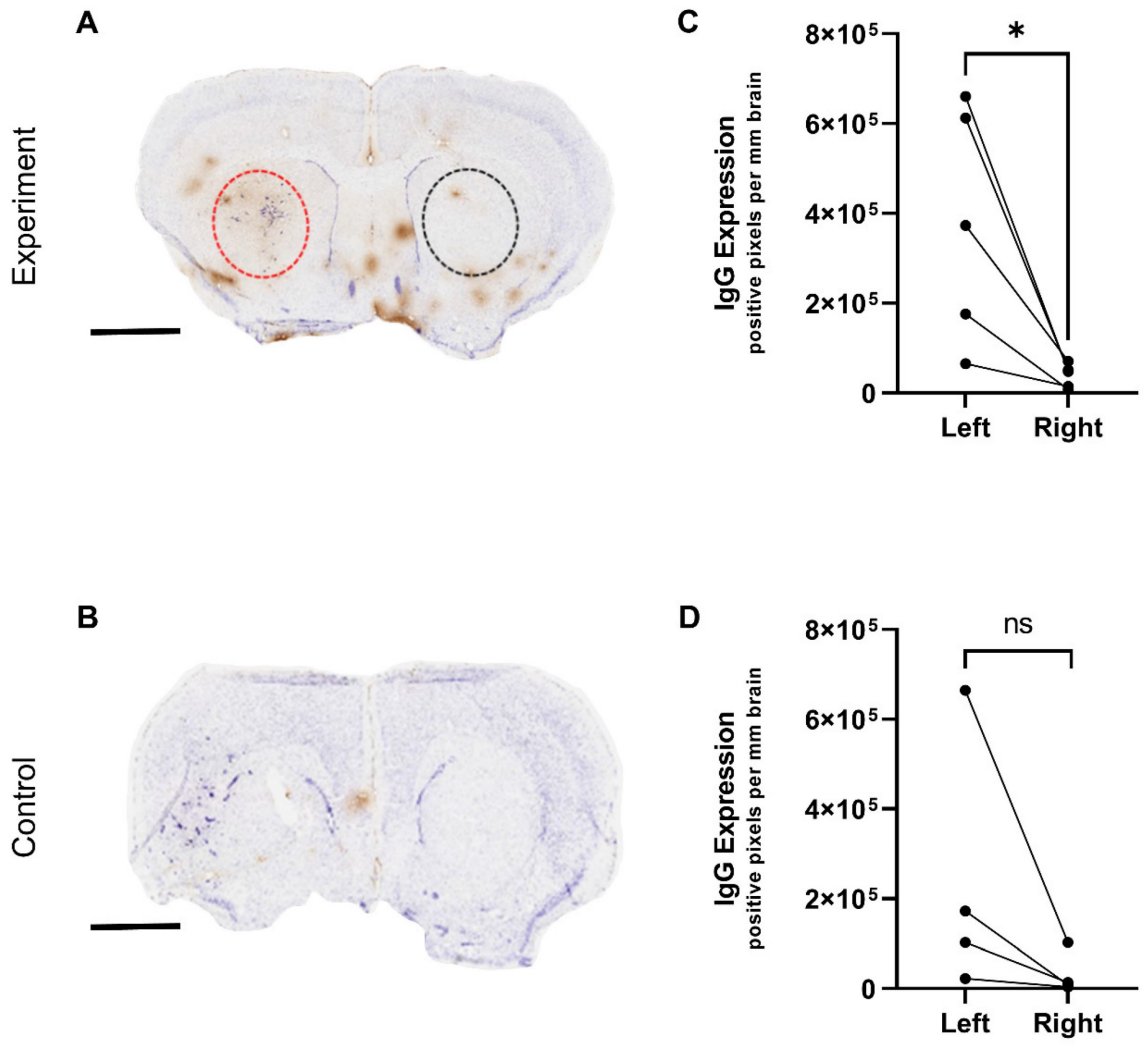
** Immunohistochemical detection of microbubble/US-induced BBB breakdown.** IgG (brown) immunohistochemistry results of mouse brain sections following AV-MB and unfocused ultrasound treatment. A) US-induced BBB breakdown was evident in animals in the experiment group as seen by brown IgG staining in contrast to reduced staining in control animals (B). IgG extravasation in the left tumour hemisphere compared to the right hemisphere was quantified and found significant in the experiment group (C) *p<0.05. The control group did not show a significant left-to-right difference (D). Sections counterstained with cresyl violet; scale bar = 2 mm.

The BBB breakdown observed by MRI from the targeted microbubbles was further assessed immunohistochemically by measuring endogenous IgG extravasation. Figure 6(A-B) shows representative examples of histology from control and experimental groups, respectively. In agreement with the MRI data, the experimental group of mice treated with targeted microbubbles and ultrasound to the brain 5 min later, showed significantly greater IgG extravasation in the left tumour hemisphere compared to the right hemisphere (Figure 6C). The control group did not show a significant left-to-right difference (Figure 6D).

A single animal in the control group appeared to have an observable left-right difference in IgG extravasation, which could be explained by pre-existing BBB breakdown prior to treatment as observed by MRI. The IgG immunohistochemistry in both groups also revealed some non-specific areas of breakdown, which again localized primarily at points of major sinuses and draining veins, where the blood flow rate is reduced and likely to cause pooling of the microbubbles in this area. This effect could potentially be avoided by increasing the time between microbubble administration and US to ensure better clearance and should be considered in future experiments.

## Limitations & Future Work

Histological examination of brain tissue was required for verification of the MR measurements. It was not, therefore, possible to investigate any post-treatment behavioural or other effects. Such assessments would, however, be extremely important in the translational development of this technique. For example, sterile inflammation has been observed in other studies of BBB opening using ultrasound and microbubbles 47. In the context of metastatic cancer therapy, we would not anticipate an extended period of repeated treatment and the risk-to-benefit ratio would likely be favourable owing to the very serious nature of the disease. Future studies must nevertheless address the question of systemic and/or long term effects of BBBD as part of a wider safety study.

The use of streptavidin and biotin in the microbubble formulation could also potentially induce negative inflammatory effects; although there are human studies using formulations containing these materials that have not reported any adverse effects 48. Streptavidin and biotin were utilised for this study as a well-established and convenient means of enabling antibody conjugation that would not negatively impact functionality. It has been shown, however, that it is definitely possible to utilise alternative conjugation methods such as a maleimide-thiol linkage or “click” chemistry methods 49. Future development of a targeted agent would certainly involve evaluation of these different methods should further *in vivo* studies indicate concerns. These studies should include quantification of appropriate inflammatory markers and pharmacokinetic studies of the microbubbles e.g. using radiolabelled drug surrogates.

By utilising the specificity of the microbubbles to localise BBB opening, rather than the physical focusing of the ultrasound, the challenges associated with compensating for the distortion of the sound field by the skull are reduced. The need to create a uniform low intensity sound field in a large tissue volume, however, presents a different set of challenges, especially when scaling up from a mouse model to a human. Recent work on ultrasound lenses could potentially be exploited to address this challenge 50,51.

Opening the BBB is a crucial step in developing effective therapies for brain cancer 9. The BBB is a protective barrier that prevents harmful substances from entering the brain, but it also blocks the delivery of potentially life-saving drugs. Opening the BBB specifically at the tumour site has the potential benefit of increasing the effectiveness of treatment, whilst minimizing side effects 52. Patients with secondary cancer in their brain have a limited time to live without effective treatment, and most drugs that we would anticipate delivering in this way only target cancer cells. Thus, any effects outwith the tumour region would be naturally limited, even in the relatively unlikely event that there was VCAM-1 expression elsewhere in the brain. This approach holds great promise not only for patients with metastatic brain cancer, but also primary brain tumours such as glioblastoma, which have recently been found to have a partially intact BBB 53, and diffuse intrinsic pontine glioma in children 54. However, it is important to note that opening the BBB can also carry risks, such as increased susceptibility to infections or other complications. As such, although the BBB opening induced here is local and focal to the tumour site, ongoing research is needed to refine this approach and ensure its safety and efficacy.

## Conclusion

The aim of this study was to investigate whether antibody targeted microbubbles could be used for localised US-BBBD. The results obtained indicate that MB functionalised with anti-VCAM-1 antibodies were able to bind selectively to stimulated brain endothelial cells expressing VCAM-1 *in vitro*; and to facilitate extravasation of both Gd-DTPA and IgG *in vivo* in the left tumour-bearing hemisphere of a mouse model of metastatic brain cancer. These findings indicate that the use of US-BBBD can potentially be extended to treat diffuse and/or early-stage disease even when tumour locations cannot be established *a priori* with conventional imaging methods.

## Supplementary Material

Supplementary figures.

## Figures and Tables

**Table 1:** Ratios of components used for the experimental microbubble formulations.

Component	Mass (mg)	Molar ratio
DBPC	14.6	90
DSPE-PEG2000-Biotin	2.7	5
DSPE-PEG2000	2.53	5
DSPE-NBD *(only added for in vitro experiments)*	0.17	1
