# Supplementary material for: Targeted opening of the blood-brain barrier using VCAM-1 functionalised microbubbles and “whole brain” ultrasound
Source: Theranostics. 2024 Jul 2;14(10):4076–89. doi: 10.7150/thno.93172 (PMC11234285; doi:10.7150/thno.93172)
Supplement: Supplementary file 1 — Supplementary figures. [file thnov14p4076s1.pdf]

SUPPLEMENTARY MATERIAL

Targeted opening of the blood-brain barrier using VCAM-1 functionalised microbubbles and "whole brain" ultrasound

Vanessa A. Johanssen[1], Jia-Ling Ruan[1], Oliver Vince[2], Alec Thomas[2], Sarah Peeters[1], Manuel Sarmiento Soto[1], Jessica Buck[1], Luca Bau[2], Michael Gray[2], Eleanor Stride[2,3] and Nicola R. Sibson[1]**

[1]Department of Oncology, University of Oxford, Oxford, UK

[2]Institute of Biomedical Engineering, Department of Engineering Science, University of Oxford, UK

[3]Nuffield Department of Orthopaedics, Rheumatology and Musculoskeletal Research, University of Oxford, UK

***Addresses for correspondence:**

Prof. Eleanor Stride, Botnar Research Centre, University of Oxford, Windmill Road, Oxford, OX3 7LD. Tel: +44(0)1865677747 email: eleanor.stride@eng.ox.ac.uk

Prof. Nicola Sibson, Department of Oncology, University of Oxford, Old Road Campus Research Building, Roosevelt Drive, Oxford OX3 7DQ. Tel. 44(0)1865677331

email: nicola.sibson@oncology.ox.ac.uk

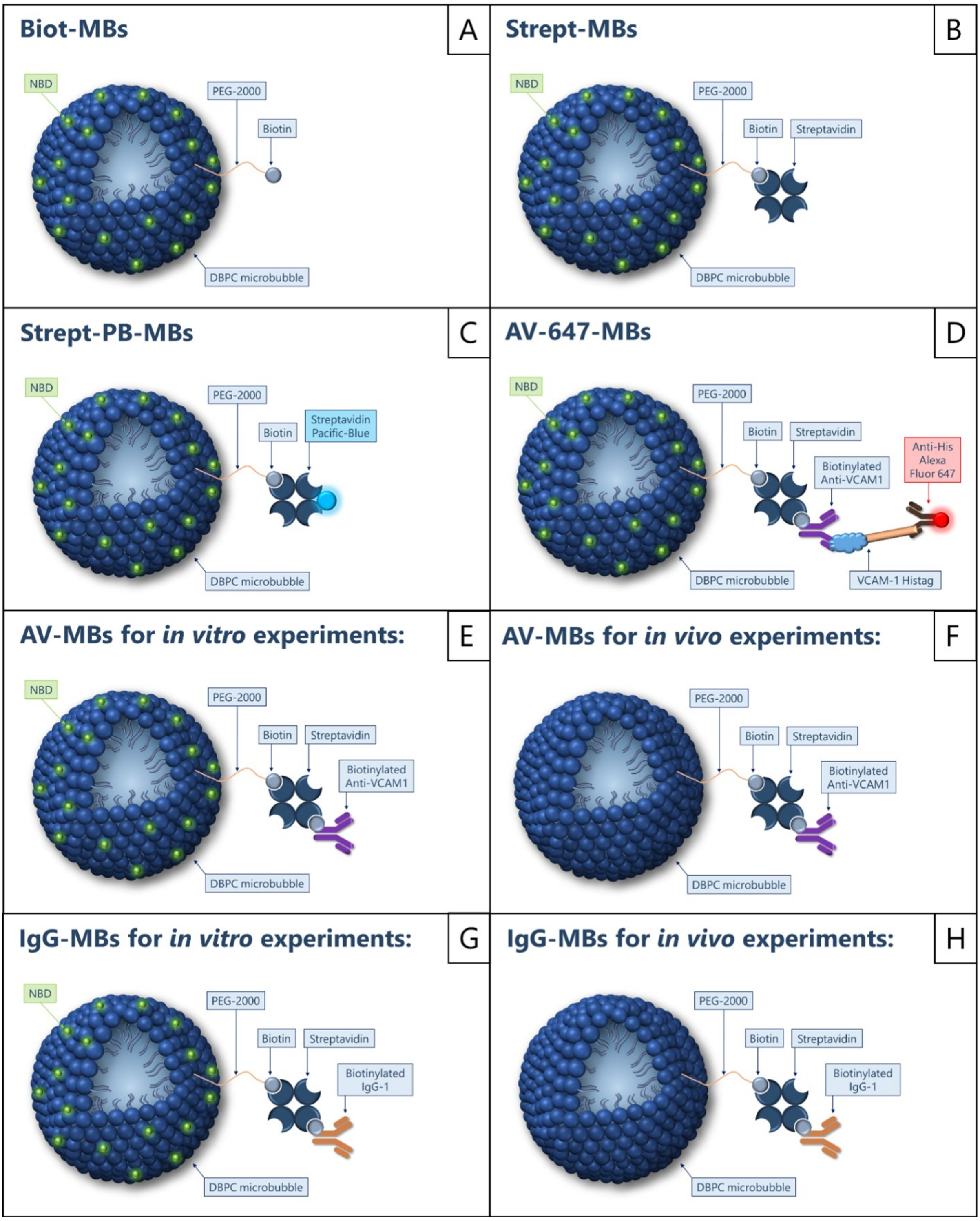

Figure S1: Schematic of the microbubble formulations used in the study. (A) Microbubbles conjugated with biotin. (B) Microbubbles conjugated with streptavidin. (C) Microbubbles conjugated with streptavidin Pacific-Blue. (D) Multi-step conjugation strategy used to verify binding of anti-VCAM-1 to AV-MBs. (E and F) Microbubbles conjugated with anti-VCAM-1 that were used for in vitro and in vivo experimentation respectively, (G), Microbubbles conjugated with IgG-1 that were used for in vitro experiments.

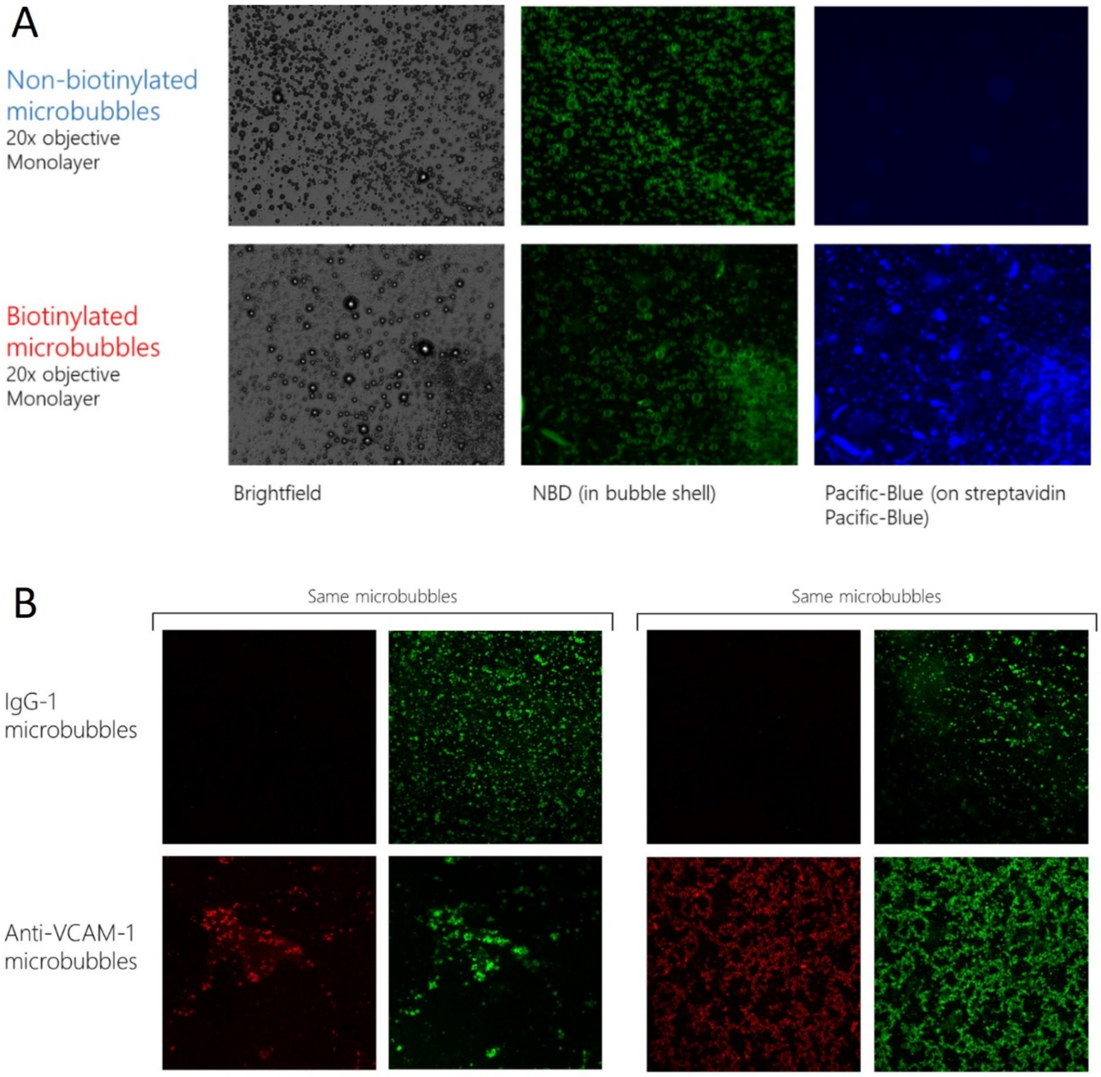

Figure S2: Microscopy images of the different microbubble formulations used in the experiments (A) Representative brightfield and fluorescence microscopy images of biotinylated and non-biotinylated MBs that were incubated with streptavidin Pacific-Blue (strept-PB) and then washed. Green shows the NBD in the microbubble shells. Blue shows the strept-PB that was only observed (after washing) on the biot-MBs. (B) Representative fluorescence microscopy images of AV-MBs and IgG-MBs that were incubated with a solution of fluorescent VCAM-1 protein. Red shows the Alexa Fluor 647 on the VCAM-1 protein and is only visible (after washing) on the AV-MBs. Green shows the NBD in the microbubble shell. Images taken at plane of maximum fluorescence intensity on a Nikon Eclipse Ti microscope. Please note that for the *in vivo* experiments, microbubbles were filtered (5 µm filter) to remove larger microbubbles.

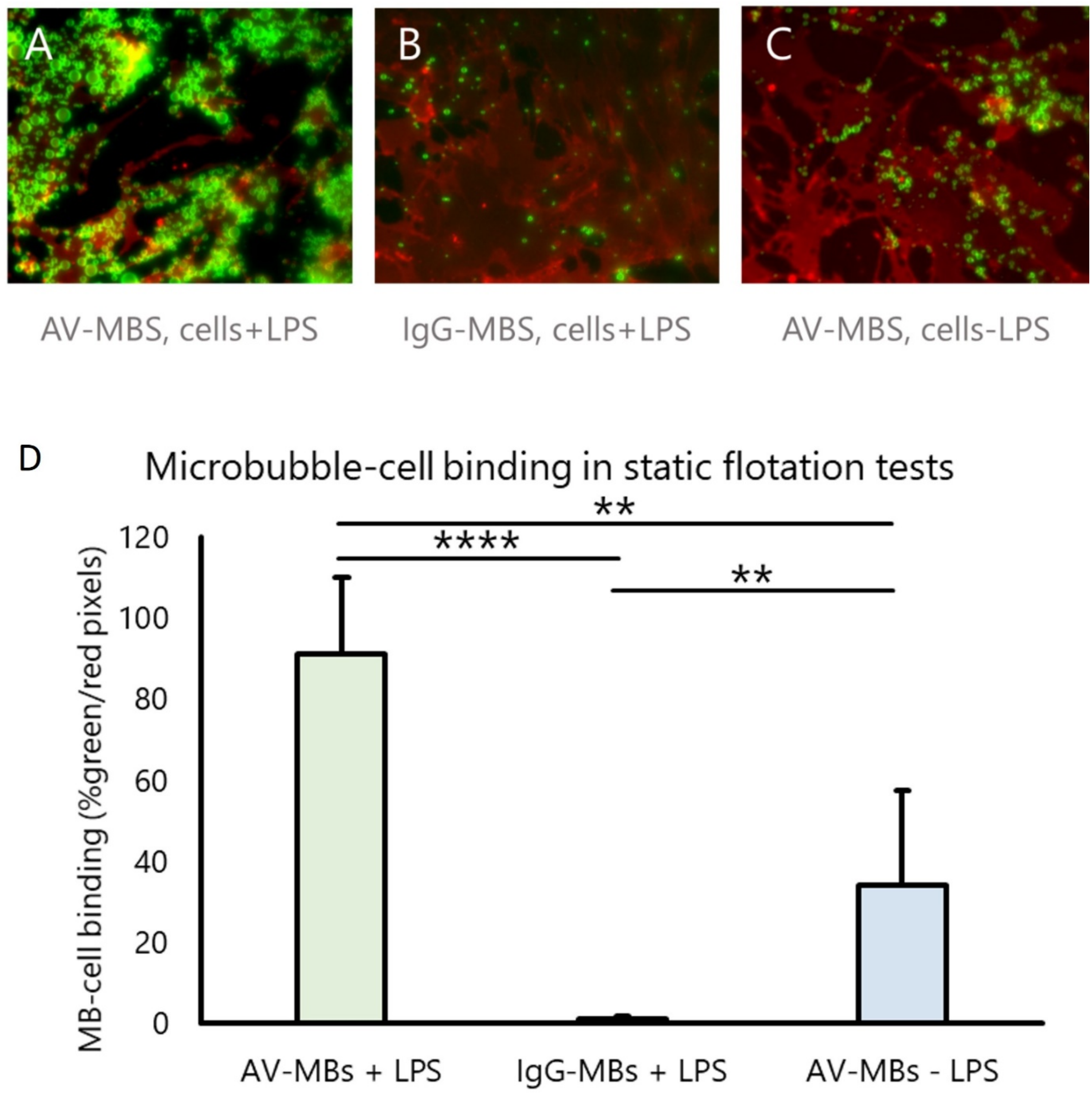

Figure S3: MB-cell binding under static flotation. AV-MBs exhibited a much higher binding to LPS-stimulated cells (A) than IgG-MBs (B). There was also some visible binding of AV-MBs to non-stimulated cells (C). (D) Graph showing MB-cell binding efficiency in static flotation tests. Binding percentage calculated by the proportion of each dual fluorescence microscopy frame occupied by microbubbles divided by the proportion of the frame occupied by cells. N = 5 per group.

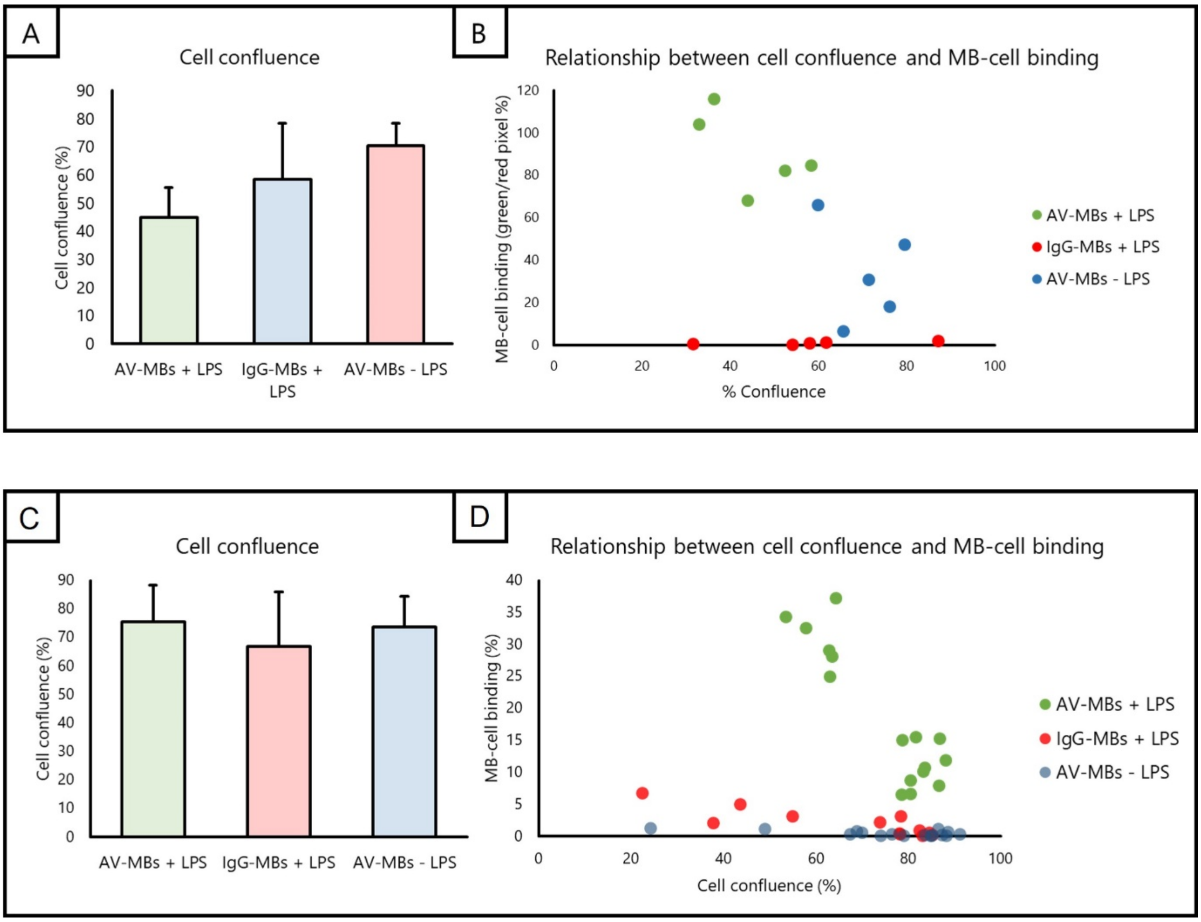

Figure S4: Effect of cell confluence on binding under static flotation and flow conditions. (A) The average confluence of the cells used in the testing of each condition under static flotation. (B) Corresponding plot showing the confluence and MB-cell binding of each sample tested. No obvious correlation is apparent. (C) The average confluence of the cells used in the testing of each condition under flow. (D) Corresponding plot showing the confluence and MB-cell binding under flow of each sample tested. No obvious correlation is apparent.

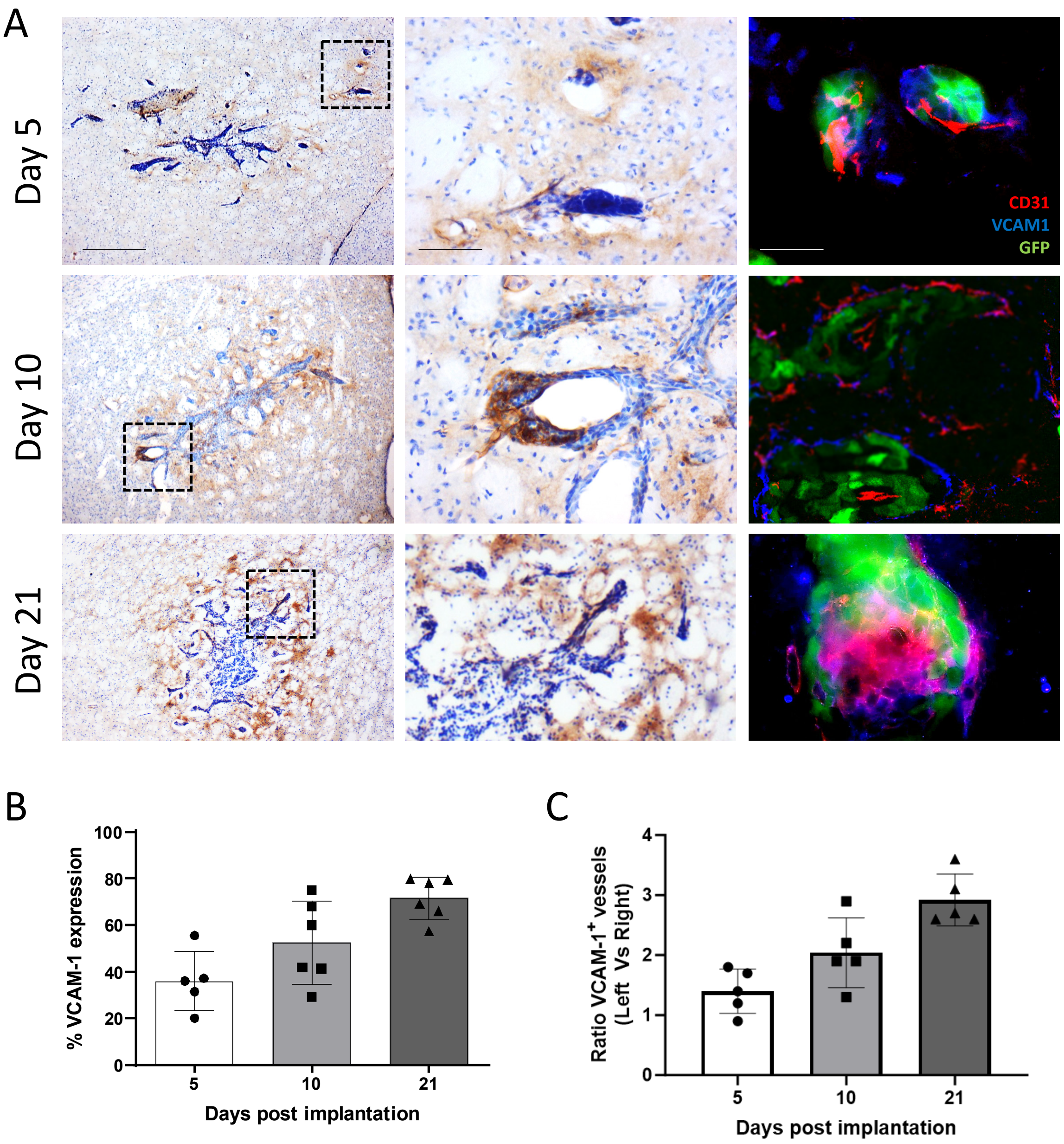

Figure S5: Quantification of VCAM-1 expression over time. A) VCAM-1 (brown) immunohistochemistry results of mouse brain sections at days 5, 10, and 21 post tumour cell implantation. Activated VCAM-1 was detected on the vessels of the left (tumour) hemisphere. Minimal VCAM-1 expression was found on the vessels of the right (control) side. Higher magnification of dotted square insets in middle column. Counterstain = cresyl violet; blue. Immunofluorescent images of corresponding magnified sections shown in right column. Fluorescent staining of VCAM-1 (blue) and CD31 (red) shows co-localisation of VCAM-1 and endothelial cells in the tumour growth area (green). B) Graph showing quantitative analysis of the percentage of VCAM-1 staining (brown in A) area within the metastatic foci. C) Graph quantitating the ration of VCAM1 positive vessels between left (tumour) and right (control) hemispheres.

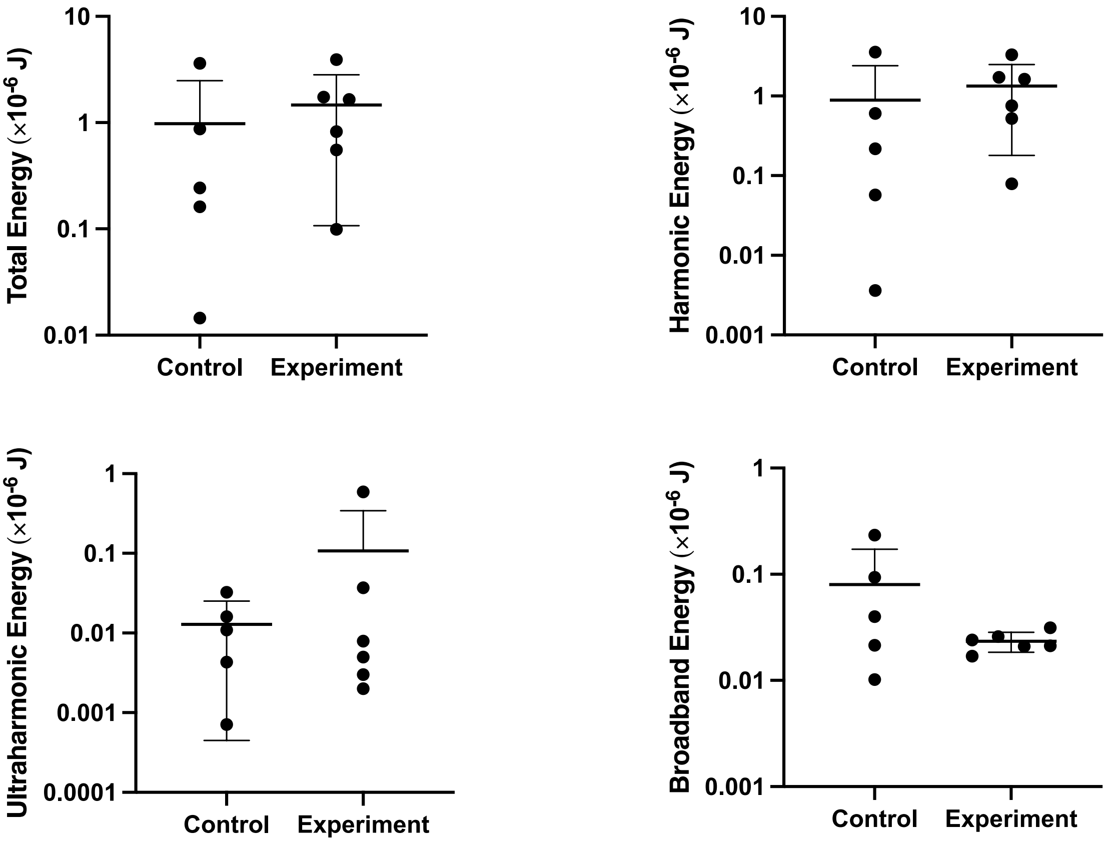

Figure S6: 5. **Acoustic emissions produced during the *in vivo* experiments.** Emissions were captured using a single element unfocused transducer (Panametrics, V382-SU, Olympus Industrial Systems Europa, Southend-on-Sea, Essex, UK) as a passive cavitation detector (PCD) with a 3.5 MHz centre frequency co-aligned with the ultrasound transducer. The harmonic and ultraharmonic signal powers were determined by integrating the power spectral density with respect to frequency over 30 kHz bands at each harmonic and ultraharmonic from 1 to 8 MHz. The broadband power was determined by integrating the power spectral density with respect to frequency over the remaining frequencies between 1 and 8 MHz. The total energy of acoustic emissions for each sample was calculated by integrating the powers (harmonic, ultraharmonic and broadband) with respect to time.
